# Comparative Experimental Study of Fracture Conductivity of Carbonate Rocks under Different Stimulation Types

**DOI:** 10.1021/acsomega.3c07319

**Published:** 2023-11-30

**Authors:** Hui Xiao, Xiaojie Xia, Chunbing Wang, Xinlong Tan, Han Zhang

**Affiliations:** Chongqing University of Science and Technology School of Petroleum and Natural Gas Engineering, Chongqing 401331, China

## Abstract

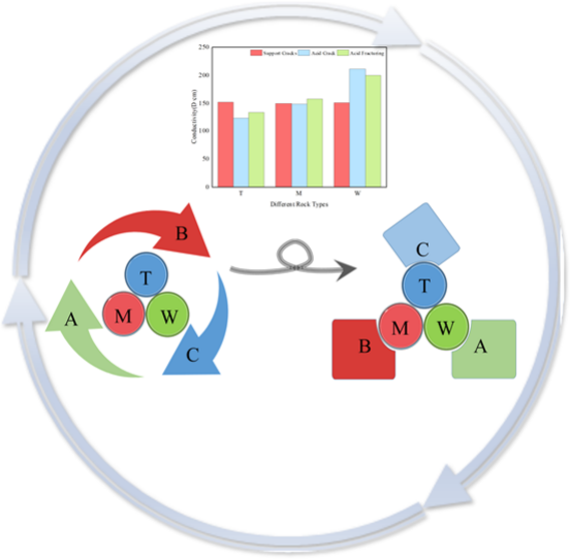

Carbonates have great potential for development, but the use of propped hydraulic fracturing or acid fracturing stimulation types alone has limited effectiveness. This study collected four sets of rock samples from three carbonate reservoirs: dolomite, limy dolomite, and limestone. The laboratory analysis focuses on propped hydraulic fracturing, acid fracturing, and acid fracturing plus proppants of these rock types. The aim is to assess acid erosion, proppant embedment depth, and the impact of varying proppant sizes and acid injection on fracture conductivity. The results showed that acid fracturing substantially enhanced the hydraulic conductivity of the three rocks. The embedded depth of the small-sized proppant was greater, but the fracture conductivity of the large-sized proppant was greater. Under the conditions of the actual pressure of formation of 55.2 MPa in the target reservoir, using 70/140 mesh proppant, 20% thickened acid, and an injection rate of 20 mL/min: for the dolomite-type rock, the propped hydraulic fracturing method had the highest fracture conductivity, which reached 151.17 D·cm. For the limy dolomite-type rock, the acid fracturing plus proppant experiment had the highest fracture conductivity, which was 157.26 D·cm. For limestone-type rocks, acid fracturing had the highest fracture conductivity of 210.39 D·cm. This study helps to elucidate the mechanism of different stimulation types and provides useful guidance for the more effective development of carbonate oil and gas reservoirs.

## 1 Introduction

Carbonates are a very important class of oil and gas reservoirs globally, and a large number of carbonate oil and gas reservoirs were discovered worldwide. For example, industrial reservoirs were discovered in the Ellenburger Group dolomite of the Lower Ordovician in the Permian Basin of North America,^1^ the Trenton and Black River Group dolomites of the Middle Ordovician in the Michigan Basin^2^ and the dolomite of the Red River Group of the Upper Ordovician in the Williston Basin.^3^ In addition, the Upper Ordovician Klasen Formation tuffs in the Baltic Sea Basin, Northern Europe,^4^ and the Middle Orbital Nita Formation dolomites in Australia’s Canning Basin.^5^ Carbonates accounted for 70% of oil and natural gas reserves, while they dominated in terms of proven recoverable reserves.^6^ Tight carbonate reservoirs were usually medium- or low-permeability reservoirs with a significant proportion of low-permeability and extra-low-permeability reservoirs. These reservoirs were characterized by significant inhomogeneities, low porosity, poor connectivity, and sensitivity to external conditions and therefore required reservoir rehabilitation.

Previously, the most common methods to improve the conductivity of tight carbonate reservoirs were mainly hydraulic fracturing and acid fracturing.^7^ Since the first “hydraulic fracturing” treatment in 1947,^8^ hydraulic fracturing was recognized as a key technology for the improvement of production rate. In traditional hydraulic fracturing, a fluid-like substance was typically introduced to formation at pressures above the normal fracture gradient. Hydraulic fracturing is a method of increasing the production capacity of oil and gas wells by applying high-pressure hydraulic forces to a rock reservoir to create fractures to increase the flow path for oil and gas.^9^ Wang’s research^10^ results showed that the granular temporary plugging agent had better plugging ability than fiber, and it was easier to plug the fractures with a small width.

As early as the 1960s, scholars began testing propped hydraulic fracturing for carbonate reservoirs. For example, Nowsco conducted fieldwork on hydraulic fracturing of carbonate reservoirs in Alberta.^11^ A systematic description of carbonate reservoirs in Texas, USA, is given by Hoover and Adams.^12^ At the beginning of the 21st century, Pinnacle conducted field trials of horizontal well-propped hydraulic fracturing of naturally fractured limestone in the North Sea.^13^ However, due to the nonhomogeneity and complex pore structure of carbonate rocks, the current fracturing design still had some deficiencies in targeting. There were complex and variable distributions of natural fractures and microfractures in carbonate rocks, and the existing fracturing software calculation model was relatively simple, which made it difficult to fully meet practical needs. Therefore, acid fracturing was recognized as a successful method to transform carbonate reservoirs. “Transform” here meant “the use of acid to dissolve some of the mineral particles or fillers in the reservoir opened or increased the fracture network, which increased the capacity of the reservoir”.^14^ Acid fracturing techniques such as large-scale, large-injection gelled acid fracturing, multistage alternating injection acid cutting, and closed acidification were commonly used techniques for carbonate reservoir reforming. Successful acid fracturing operations helped to reduce formation damage and increase productivity. An important factor in realizing nonuniform acid etching on acid fracture surfaces was the nonhomogeneous nature of mineral distribution in the reservoir.^15^ Carbonate reservoirs were highly inhomogeneous and contained many acid-insoluble materials that played a supportive role when they were subjected to closure stresses.

Fracture conductivity is one of the most important parameters for evaluating the effectiveness of fracturing. In a complex fracture network, economic production would not be achieved if sufficient fracture conductivity could not be maintained. In 1998, Navarrete’s study^16^ found that by applying high pressure, rocks would form cracks, and these cracks would expand. The ability of the fracture to provide a high conductivity depended on both the fragmentation and the embedding of proppant particles. In 2005, Weaver^17^ further discussed that proppant embedment in the formation became a critical factor when fracture closure pressures were high, particularly for hard formations such as sandstones and carbonates. Sandstone and carbonate formations were typically highly porous and permeable, and proppant could fill and solidify formation pore-supported fractures as well as reduce fluid flow in the pores, which could lead to a significant reduction in hydraulic conductivity.

Scholars have carried out a great deal of research centered on improving the fracture conductivity of acid-eroded fractures. Beg^18^ conducted systematic testing of acid-etched fracture conductivity and pointed out that deep grooves on acid-etched fracture surfaces could provide a large conductivity, but overall fracture conductivity was also related to the surface stresses after acid etching. Wang et al.^19^ found that with the increase in injection stages, the corrosion ability of acid to fractures was significantly enhanced. Malik^20^ designed a test for acid-etched fracture conductivity and described for the first time the effect of acid-etched fracture surface on the conductivity. Ruffet et al.^21^ studied the effect of shape (e.g., morphological features such as depressions, protrusions, cracks, pores, etc. on the surface of the fracture surface) and roughness of the fracture surface on conductivity after acid-etching using a large core as a research object. They proposed an experimental methodology to investigate and characterize quantitatively how acid injection conditions affected the fracture surfaces, how the rough surface was able to support the stress, and finally how fracture conductivity could be estimated from the surface topography. Wang and Lv^22^ used composite stimulation technology for 9 wells in carbonate and shale oil and gas reservoirs. This technique achieved good stimulation effects and increased production by 210% higher than that of gelled acid fracturing after fracturing. Dong^23^ and others presented a methodology for predicting the propagation of acid along natural fracture networks and the resulting etching of the fracture walls. They used roughness mathematical methods to characterize the surface morphology of acid-etched cracks and calculate the fracture conductivity of the acid-etched cracks.

Jones^24^ investigated the variation of flow in carbonate reservoirs under closure pressure. They found that the pressure of higher closure resulted in lower fracture conductivity. The study by Akbari et al.^25^ found that the mechanical strength of the rock at the seam face had a significant effect on the fracture conductivity of acid-fractured fractures, with stress of higher closure leading to a greater impact of the strength of rock mechanical on fracture conductivity. Malik and Hill^26^ and others independently developed an experimental testing apparatus for the fracture conductivity of acid-fractured cracks, taking into account the influence of morphological characteristics of the fracture surface on fracture conductivity for the first time. Bartko et al.^27^ conducted acid-etching experiments on different carbonate rocks and investigated changes in morphological characteristics of the incised surface and fracture conductivity under the influence of various types of acid solutions. This study indicated that optimal treatment could be achieved through a combination of emulsified acid in a fracture acidizing treatment followed by a closed fracture acidizing stage using emulsified acid, which led to increased productivity in Lisburne Field. Van et al.^28^ found through experimental research that nonuniform etching morphological characteristics of the seam surface were an important factor affecting the fracture conductivity of acid fracturing, but fracture conductivity was not absolutely proportional to the volume of acid etching. Wang^22^ suggested a compound acid fracturing method, which first used proppant to prop up the tip of the fracture, and then used gelling acid to corrode the middle and rear sections of the fracture, effectively improving inflow capacity.

Melendez et al.^29^ studied acid-etching experiments on chalk, dolomite, and chert and found that increasing reaction time and temperature did not consistently improve conductivity. Pournik et al.^30^ experimentally investigated the effect of different acid solutions on the fracture conductivity of acid fracture and recognized the effect of acid type and concentration on fracture conductivity. The experimental results showed that viscoelastic acid generated the highest conductivity at the stress of low closure and emulsified acid resulted in the largest retained conductivity at higher loads. Neumann et al.^31^ experimentally demonstrated that the fracture conductivity of elastic fracture mainly depended on the spacing between the “concave” and “convex” surfaces formed by acid etching. Al-momen et al.^32^ used dolomite cores to study the effects of contact time, acid type, and temperature on fracture conductivity. Under relative stress of low closure, the fracture conductivity of a rough fracture surface was significantly higher than that of a smooth fracture surface.

Fojtasek^33^ concluded from his study that limestone reacted favorably to being acid-fractured at low acid residence times and yielded conductivity values higher than those of a propped fracture. Mehrjoo et al.^34^ evaluated the effects of rock strength, injection rate, acid type, and closure stress on ultimate fracture conductivity and developed three models for estimating ultimate fracture conductivity. Hassan^35^ found that significant increases were observed in the fracture conductivity for chalk and limestone rocks when treated with GLDA, while a slight increase was achieved for the dolomite conductivity. Likewise, Tariq et al.^36^ experimentally provided new insights into the selection of the acid fracturing fluid for different carbonates, and less reactive rocks such as dolomites were not good candidates to be treated with chelating agents because of their low reactivity.

Calcite rocks (i.e., limestone and chalk) can be treated with chelating agents or strong acids. Kong et al.^37^ concluded from the study that for low-permeability carbonate reservoirs, the acid sand-carrying fracturing technique was more reliable than the simple acid fracturing technique and achieved better stimulation and conversion effects.

Although a large number of studies have been conducted on hydraulic fracturing and acid fracturing of carbonate rocks, there have been relatively few studies on fracture conductivity due to different fracturing methods in the same carbonate reservoir. Therefore, it has been necessary to study different fracturing methods and their fracture conductivity in different types of carbonate rocks. This study has focused on carbonate formations, using several carbonate mineralogies, which have been limestone, limy dolomite, and dolomite. Several different stimulation types have been designed for acid fracturing, propped hydraulic fracturing, and integrated design of acid fracturing plus proppant, which have been the techniques we have used for evaluation in order to gain a deeper understanding of the changing law of fracture conductivity in carbonate reservoirs and to provide new ideas for carbonate reservoirs evaluation.

## 2 Methodology

This section outlines how the samples were prepared for fracture conductivity testing and the associated procedures and equipment used. In order to obtain propped hydraulic fracturing fracture conductivity closer to actual conditions, actual formation cores were used to make standard API rock slabs, and the slabs were used to carry out fracture conductivity tests considering proppant embedding. The proppant embedding and fracture conductivities of different carbonate rocks were analyzed.

Twelve carbonate rock core samples of dolomite, limy dolomite, and limestone were tested. One test was first performed using 40/70 mesh ceramic and 70/140 mesh ceramics filled separately. A second test was conducted using 20% thickened acid with an exposure time of 30 min, after which the core samples were tested again. In the third test, the acid-etched plates were sand-filled and then filled with 40/70 mesh ceramic and 70/140 mesh ceramic, respectively, and tested again.

### 2.1 Experimental Material

#### 2.1.1 Sample Selection

##### 2.1.1.1 Sample Selection

The mineral content determined the selection of samples, which included four T-group dolomite samples with calcite, four M-group limy dolomite samples featuring calcite and dolomite, and four W-group limestone samples. An X-ray diffractometer was used to analyze the mineral constituents of different carbonate rocks to obtain the main mineral compositions and contents. The compressive strength of 12 groups of samples was also tested.

**Figure 1:**
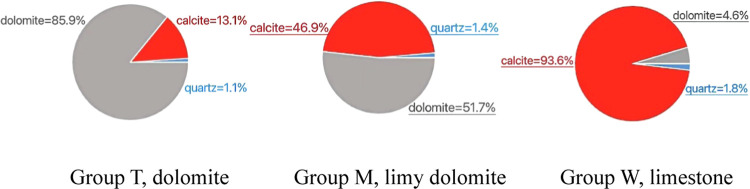
Average of three sets of samples from X-ray diffraction analysis of carbonate outcrop.

**Figure 2:**
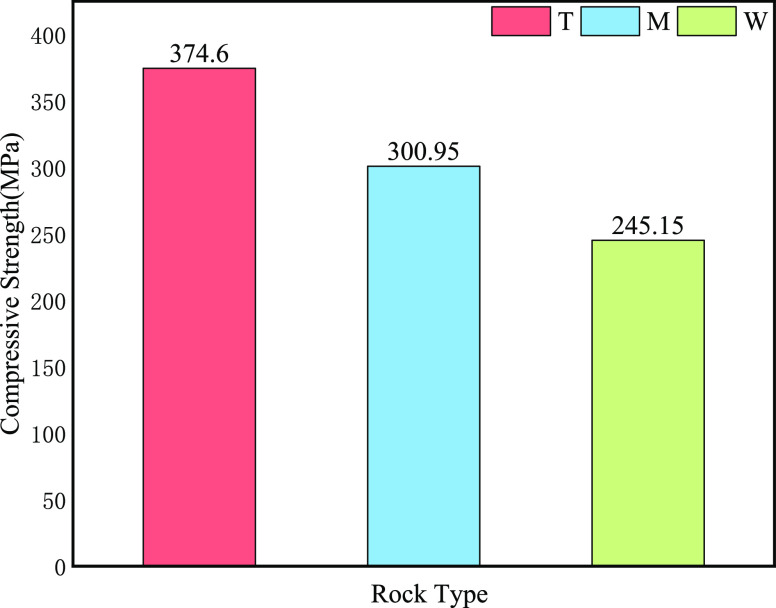
Average compressive strength of three groups of samples.

A total of 12 groups were analyzed by X-ray diffraction (XRD). Four sets of each of the three different lithological cores (divided into T, M, and W sets) were selected, and three rock compositions were measured in each set. An average value was taken for each composition of rock in each of the four sets, and from these data, the values of the final average in Figure 1 were obtained. Group T had more than 85% dolomite, over 51% in dolomite group M, and over 93% calcite in group W. The mineral composition of the three groups was mainly calcite and dolomite with very little quartz. Meanwhile, in Figure 2, a total of 12 groups were measured for compressive strength. To make this measurement, four sets of each of the three different lithologies of the cores (divided into T, M, and W groups) were selected and an average value was taken for each of the four sets. The compressive strength of group T was 374.6 MPa (the highest strength), the compressive strength of group M was 300.95 MPa, and the compressive strength of group W was 245.15 MPa (the lowest strength).

##### 2.1.1.2 Processing Rock Samples

**Figure 3:**
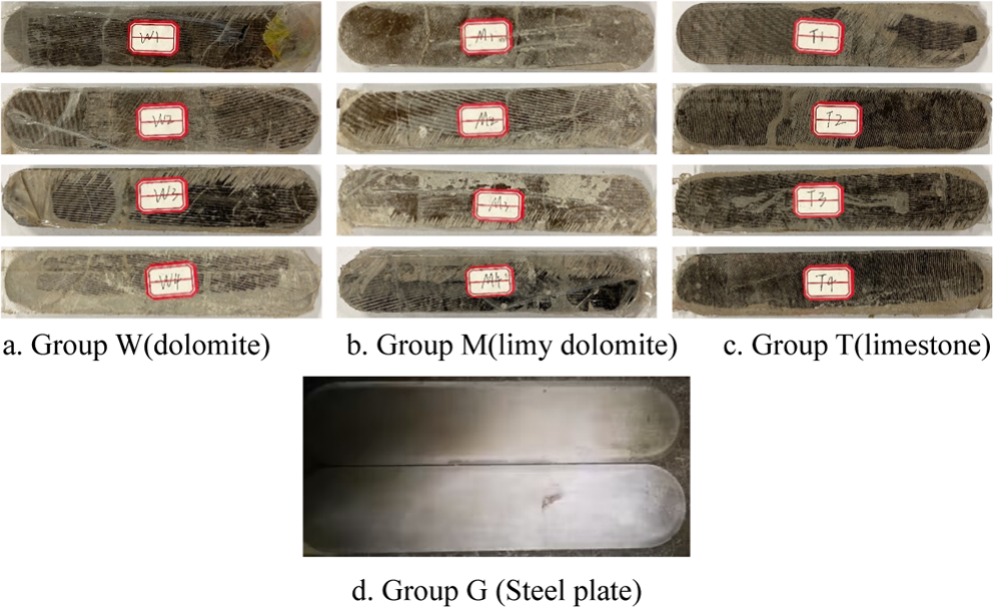
Two steel plates and 12 samples from three different lithological carbonate rocks (Photograph courtesy of Xiaojie XIA. Copyright 2023).

The rock samples were processed into a rock sample with a rectangular shape in the middle and semicircular arcs at both ends so that the shape of the experimental samples matched the shape of the API infiltration chamber. Full-size cores and professional equipment were used to produce an API standard rock plate with a length of 174 mm, a width of 34 mm, a thickness of 10 mm, and a radius of the arc of 1.7 cm. The propped hydraulic fracturing experiments used steel plates, which were also API standard and had the same dimensions as the rock plates. Photographs of the steel plates and rock plates are shown in Figure 3.

##### 2.1.1.3 Rock Plate Packaging

In order to ensure the sealing needs of the experiment, the rock samples were stuck together and then wrapped. The wrapping material was the backing. In this case, cracks in the rock samples were prevented from forming in the glue. Before the configured perfusion gel was placed in the incubator, all rock samples were wrapped to ensure that the gel could solidify stably. The rock sample had to be extracted only once to get rid of any leftover cement, and the two samples were then combined to reveal the overacid fracture surface.

#### 2.1.2 Proppant Selection

**Figure 4:**
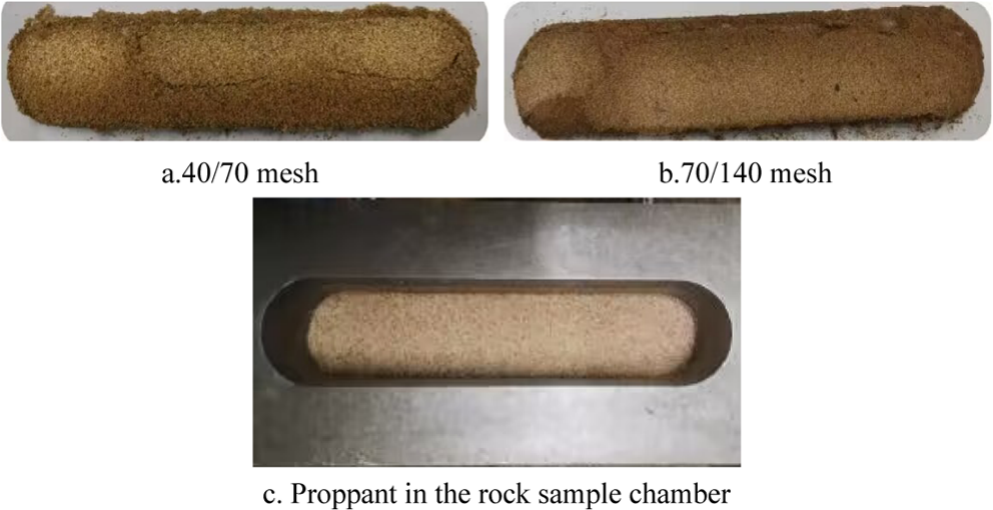
Pictures of the two proppants used in this experiment and before the proppants were placed in the rock sample chamber for the experiment. (Photograph courtesy of Xiaojie XIA. Copyright 2023).

According to the site implementation, 40/70 mesh and 70/140 mesh ceramic proppants were selected to carry out the test. Combined with the pressures of closure of the layers at the reservoir site, the pressures of closure set in the laboratory ranged from 6.9 to 55.2 MPa. The proppant concentration in the field was low, resulting in an experimental test with a proppant concentration of 5 kg/m^2^ for sand. Pictures of the two proppants used in this experiment are shown in Figure 4(a,b). The process of adding proppant in the rock sample chamber in Figure 4(c) is also shown.

#### 2.1.3 Acid Selection

In order to more accurately simulate the acid–rock reaction during acid fracturing in the field, a thickened acid solution was used for the laboratory acid-etching experiments. The formula for the acid solution was 20% HCl + 0.2% CJ1–3 (A)+1.0% CJ1–3 (B). Materials of specific experiments were selected as shown in Table 1.

**Table 1:** Specific Experimental Protocols for Propped Hydraulic Fracturing

conditions of the experiment	specific values
rock type	T1/T2: dolomite; M1/M2: limy dolomite; W1/W2: limestone; G1/G2: steel plate
stimulation type	propped hydraulic fracturing, acid fracturing, and acid fracturing plus proppant
pressure of closure	6.9–55.2 MPa
particle size of proppant	40/70 mesh and 70/140 mesh
proppant concentration	5 kg/m^2^
media for testing	nitrogen

### 2.2 Experimental Setup

**Figure 5:**
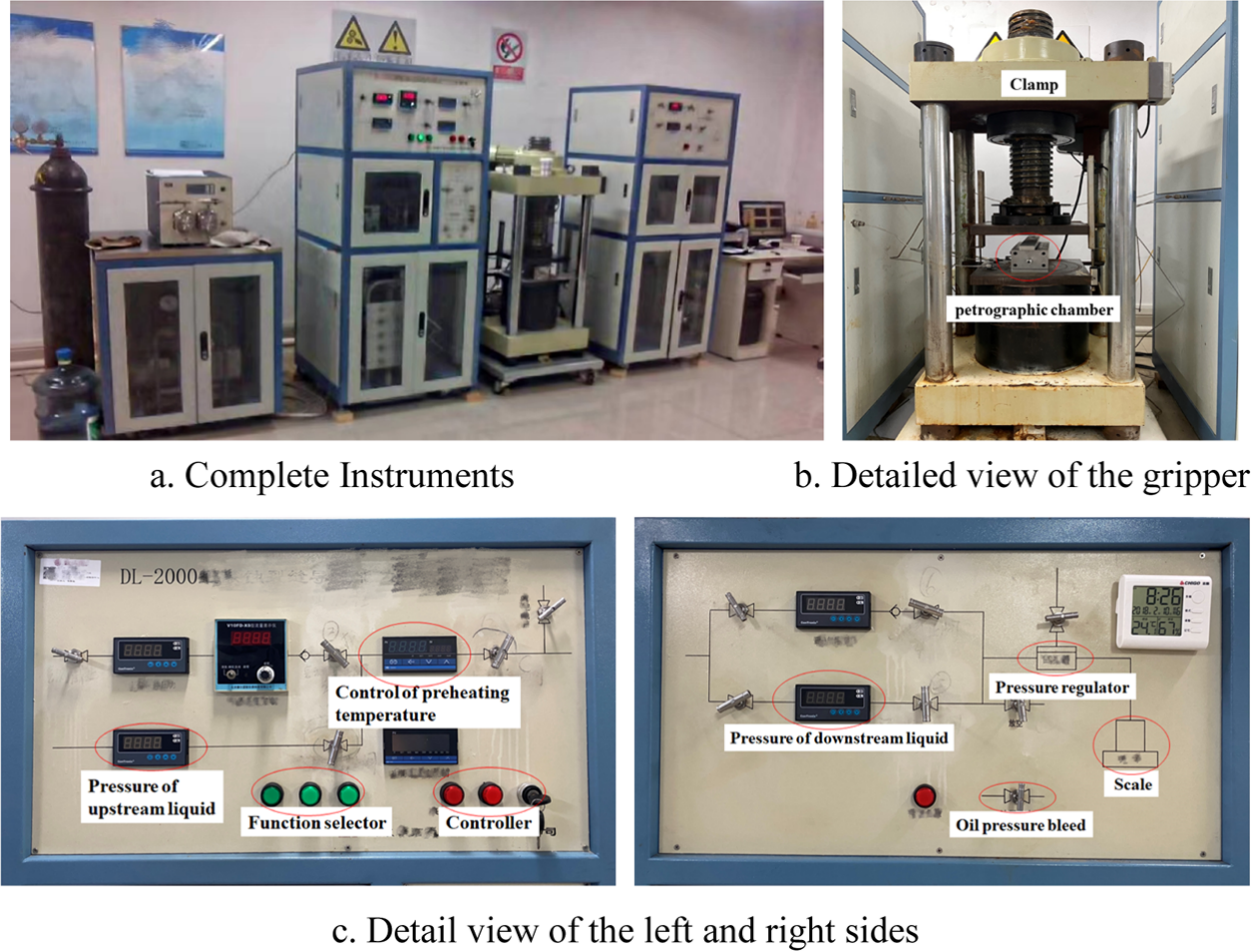
Instruments used in the experiment: acid-etched crack conductivity tester (DL-2000) setup in the laboratory (Photograph courtesy of Xiaojie XIA. Copyright 2023).

The experimental setup shown in Figure 5 was used for this experiment. An acid-etched fracture conductivity tester (DL-2000) was used for proppant crushing, acid-etched fracture experiments, and conductivity testing. This conductivity tester could carry out the test for fracture conductivity with a temperature of 150 °C, a pressure of closure of 60 MPa, and a pressure of flow of 40 MPa, which satisfied the demands of this experiment.

### 2.3 Experimental Program

#### 2.3.1 Propped Hydraulic Fracturing

##### 2.3.1.1 Experimental Program

According to the site conditions, 70/140 mesh and 40/70 mesh proppants were selected for testing. The test groups were categorized into 1 and 2. One experiment was conducted for each rock slab. The pressure range of experimental closure of 6.9–55.2 MPa was determined based on the pressure of closure of each layer. The values of 6.9 13.8, 27.9, 41.4, and 55.2 MPa closure stress points were recorded. The embedment depth of the proppant at 55.2 MPa was tested with emphasis. Considering the low concentration of proppant, the concentration of sand laid during the test was 5 kg/m^2^ and the test medium was nitrogen. The experiment was conducted in accordance with the “Test Method for Fracturing Proppant Conductivity” SY/T 6302–2019 standard.

The embedding depth of the rock slabs after carrying out the embedding tests was tested using a body microscope. The embedding depth was calculated by combining the geometric features of the spheres. The embedding depths of different carbonate rocks could be obtained in this way.

“*h*” is the embedment depth for the single rock slab test. “*h*_1_” and “*h*_2_” are the embedment depths of the upper and lower rock slabs, respectively. “*R*” is the radius of the proppant particles. “*L*” is the length of the proppant embedment groove. In this paper, a value of 0.3 mm was taken for the radius of the particles “*R*” for 40/70 mesh proppant, and a value of 0.15 mm was taken for the radius of the particles “*R*” for 70/140 mesh proppant. The value of “*L*” was the length of the proppant embedded in the groove as actually measured after the specific experiments. The value of “*H*” was obtained by the formulas of “*R*” and “*L*”. The value of “*L*” was the length of the proppant embedded in the groove as measured after the specific experiments. The final results are presented in Section <span class="spnCursor"></span>2.3.5.1.1.
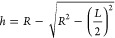
1

##### 2.3.1.2 Experimental Procedure

**Figure 6:**
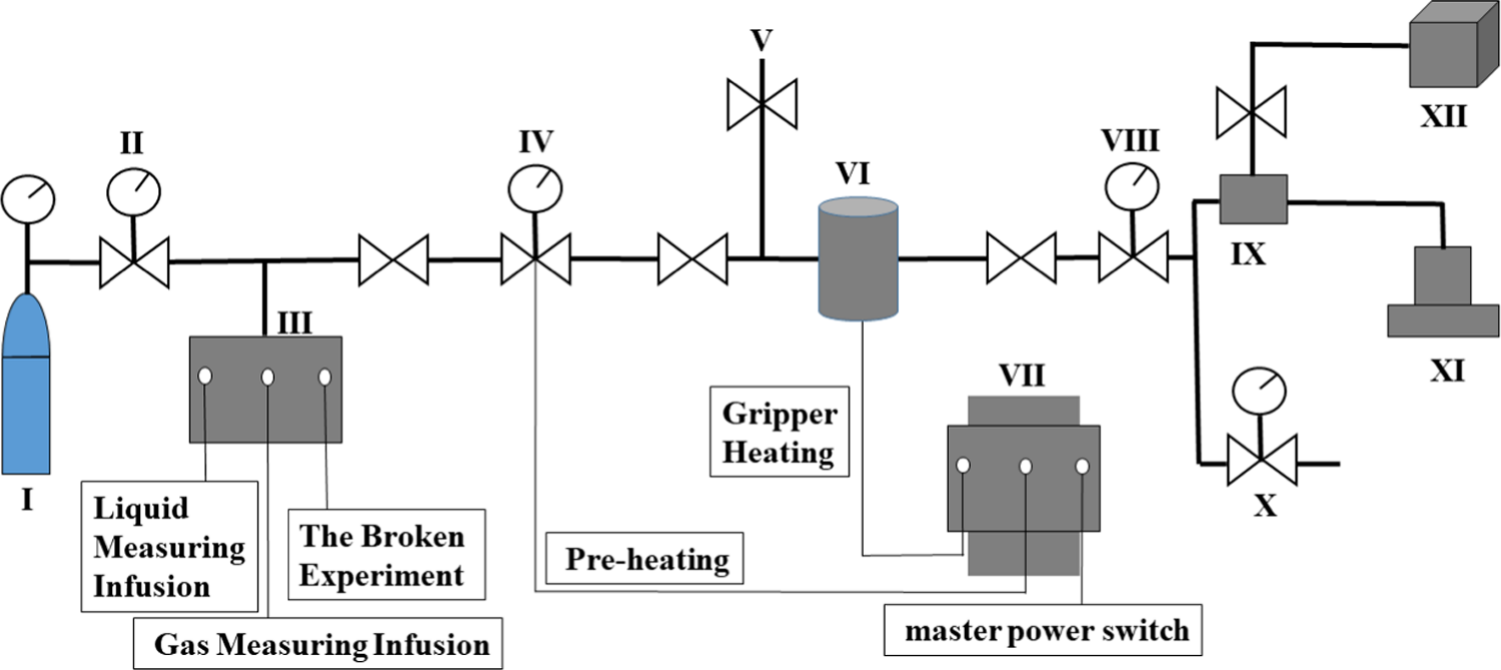
Experimental schematic for measuring the fracture conductivity.

The crack infusion process was as follows: silicone rubber was applied, infusion chambers were installed, the crack was aligned, and an inlet/outlet was set up. The hydraulic press was boosted to the designed closing pressure and stabilized for 5 min. The matching flapper was installed to ensure a horizontal contact. The vacuum pump was turned on and exhausted for 30 min to saturate the deflector chamber and piping with fluid. A displacement transducer was installed to record crack changes at different pressures in the test. Inputs were set as parameters to start the fracture conductivity testing system. The flow rate of the advection pump was adjusted, and data was recorded. Steps were repeated with increasing closure pressure, and data was recorded at different pressures. The equipment was turned off, and the inflow chamber and the rock sample were disassembled. The data were processed to derive the fracture conductivity of fractures at different pressures of closure. The specific experimental schematic of the fracture conductivity is shown in Figure 6.

#### 2.3.2 Acid Fracturing

##### 2.3.2.1 Acid Etching Experimental Program

The experiment was mainly conducted to determine the acid etching of different carbonate rocks under field conditions, including the T group limestone, the M group limy dolomite, and the W group dolomite. An acid etching fracture conductivity tester was used for the acid etching experiments on rock slabs. The program of specific experimental is shown in Table 2.

**Table 2:** Specific Experimental Protocols for Acid Fracturing Experiments

conditions of the experiment	specific values
rock type	T1/T2: dolomite; M1/M2: limy dolomite; W1/W2: limestone
composition of the acid fracturing liquid	20% HCl +0.2% CJ1–3(A) +1.0% CJ1–3(B)
temperature of acid fracturing	106 °C
the volume of the liquid	120 mL
reaction time	30 min
discharge volume	15 and 20 mL/min
pressure value	7.6 MPa
acid concentration	20%

##### 2.3.2.2 Acid Etching Experimental Program

The method used for acid etching experiments involved placing rock samples in a sampling chamber that was completely sealed and then immersing them in brine. To exclude gas and ensure saturation, we treated the samples with a vacuum pump in a vacuum environment. Silicone rubber was applied on the rock plate, and a 1 mm thick gasket was used to control the width of the seam. Silicone rubber was also used to seal the seam and prevent damage to the rubber ring. Flappers were installed in the inlet and outlet of the infusion chamber to ensure horizontal contact and prevent the leakage of liquid. The acid etching thermostat box was then installed, the piping was connected, and the seal was checked. The acid solution was prepared, gas was exhausted, and the acid-proof gears were worn. A clean water antileakage test was conducted, and any leakage was observed. The preheater was adjusted to the required temperature. The pipeline was emptied; experimental parameters were entered, and the program was initiated. The valve at the end of the loss of filtration was opened; the back pressure was adjusted, and the loss of acid was collected. The valve of the acid tank was opened, and the valve of the water tank was closed. The acid was pumped according to the set displacement and stability, and any leakage of acid was observed; the infusion chamber was flushed after completing the test. The pump was turned off, and the experiment was ended. The infusion chamber was dismantled, and the equipment was cleaned. The experimental program was stopped, and the data was exported.

##### 2.3.2.3 Laser Scanning Experimental Program

Scans were performed on the rock slabs following propped hydraulic fracturing experiments. Using a unique spray, we then applied the nonreflective solution on seam surfaces and allowed it to dry without any reaction. The nonreflective solution on the slabs was then cleaned up with pure water. The rock samples were then subjected to acid etching experiments and scanned. The acid-etched rock slabs with an added proppant were also scanned. Finally, the three-dimensional (3D) data obtained from the scans were organized and used to calculate a variety of quantitative parameters to characterize the surface morphology.

##### 2.3.2.4 Acid Fracture Conductivity Program

The rock slabs were used for the fracture conductivity test of an acid-etched fracture after the acid-etching experiment described in the previous section. Referring to Section 2.3.1, the propped hydraulic fracturing fracture conductivity test was utilized as the experimental scheme and procedures used.

#### 2.3.3 Acid Fracturing Plus Proppant

In that experiment, acid-etched rock slabs were used and sand was filled into them afterward. The acid-etched crack conductivity tester was used to carry out the experimental testing of the fracture conductivity of acid-etching and acid fracturing plus proppant under different pressures of closing and to obtain the fracture conductivity under the corresponding conditions. The study utilized the same experimental apparatus, protocol, and procedures as in Section 2.3, propped hydraulic fracturing fracture conductivity experiment.

#### 2.3.4 Experimental Considerations

**Figure 7:**
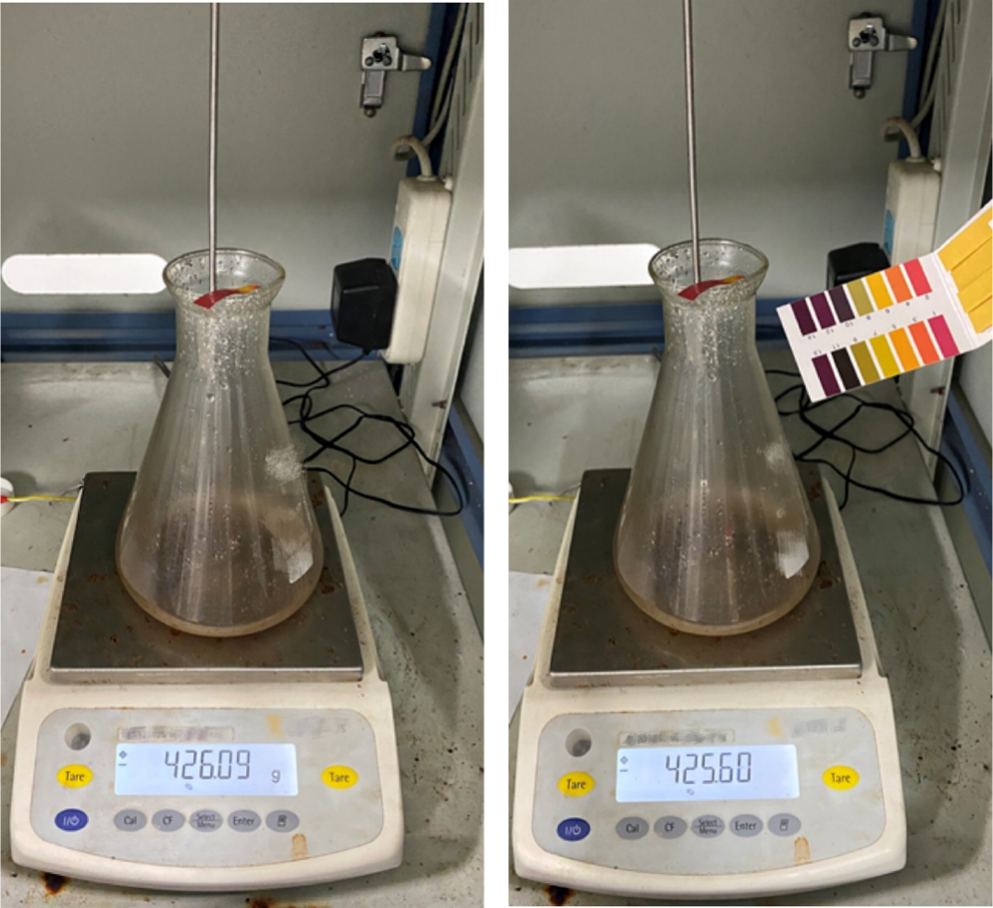
Schematic diagram of the process of testing the pH value of the acid before cleaning the equipment (Photograph courtesy of Xiaojie XIA. Copyright 2023).

Since the acid etching experimental process injected corrosive acid into the crack under high temperature and high-pressure conditions, the experimental operation had to ensure safety. The pH was adjusted to a value of the neutral range prior to cleaning to ensure safety (Figure 7). Protective coverings, goggles, protective clothing, and shoes were worn during the experiment. Other safety measures such as fire extinguishers, chemical safety rules, and acid-filled containers were prepared. Also, to maintain equipment pressure and prevent leaks, the fittings were coated with epoxy and tightened. Squeezing the sample could result in uneven distribution of the proppant and affect the test results. Uniform sample, test, and proppant distribution were required for accurate comparison of results. Tape was used to cover and smooth to prevent leaks. Nitrogen flow was monitored during test tank pressurization to prevent the proppant blowout.

## 3 Results and Discussion

### 3.1 Propped Hydraulic Fracturing

#### 3.1.1 Proppant Embedding

**Figure 8:**
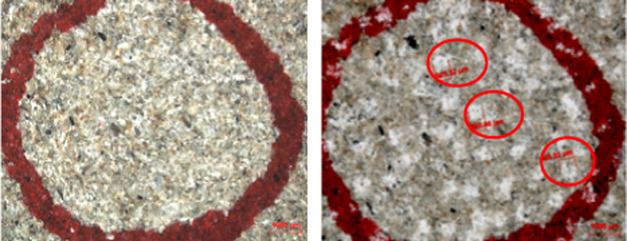
Microscopic imaging before (left) and after (right) embedding of the support agent.

**Figure 9:**
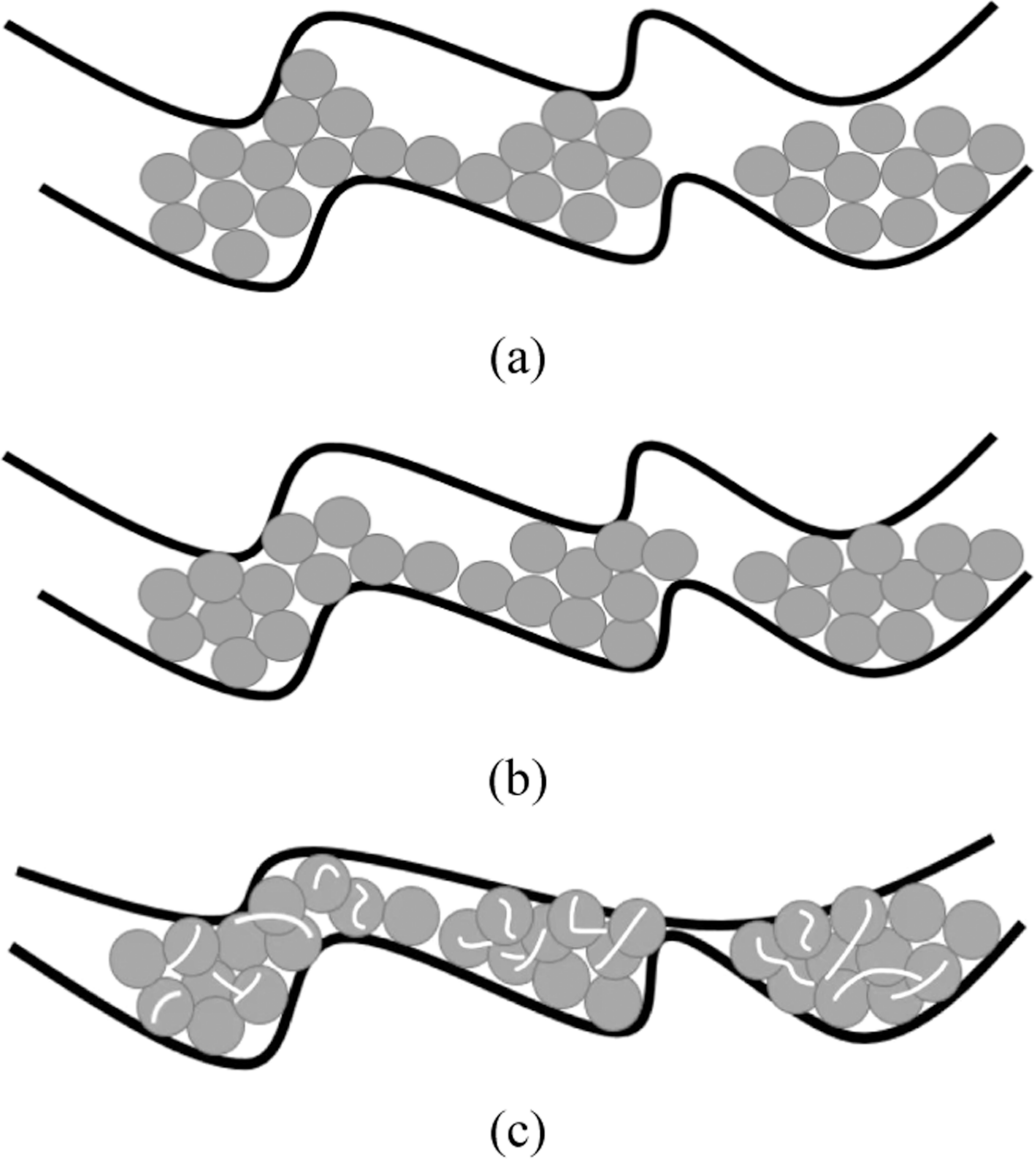
Proppant distribution on a rough rock fracture surface: (a) initial proppant pack, (b) proppant rearrangement during the application of closure stress, and (c) proppant crushing as the rock creeps at high closure stress.

In addition to the embedment depth of the proppant that affected the cracks, the proppant produced crushing and elastic deformation under pressure conditions that also caused crack deformation, as shown in Figures 8 and 9. The embedment of the proppant was combined, and the analysis was expanded together in the conclusion.

**Figure 10:**
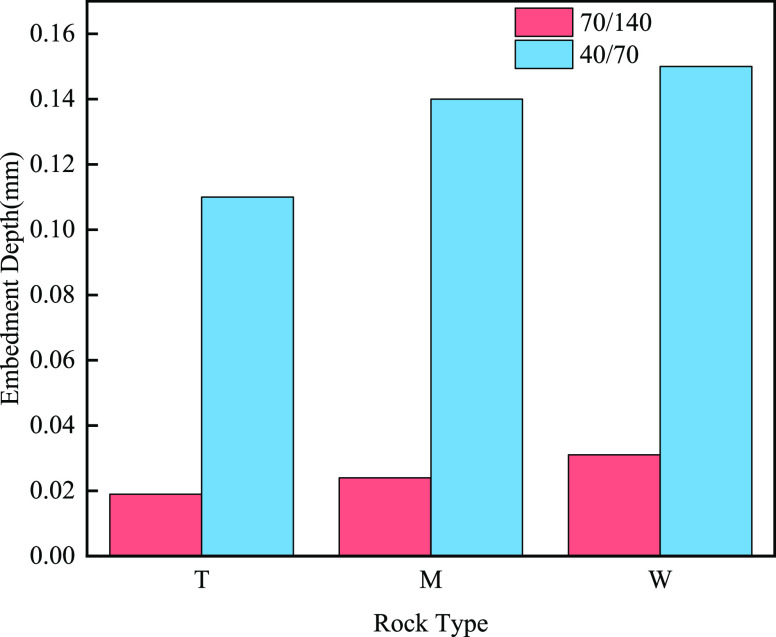
Embedding depth of proppant with different particle sizes in different layers at a pressure of 55.2 MPa.

For each rock type, there were four rocks divided into two groups: test groups 1 and 2. For each group of rocks, different proppant experiments were conducted, and the average of the two taken was the final data. One group used 40/70 mesh proppant, and the other group used 70/140 mesh proppant. Both used a single layer of proppant. The results were as follows: Figure 10 and Table 3 show that the proppant embedding depth was larger using 70/140 mesh size, but the fracture conductivity was lower than the case of using 40/70 mesh size. The rock strength of dolomite in group T was the highest at 374.6 MPa, but the lowest depth for proppant embedding was 0.11 mm (70/140 mesh). The rock strength of limestone in group W was the lowest at 245.15 MPa, and the highest depth for proppant embedding was 0.15 mm (70/140 mesh). Because limestone typically had a high solubility and permeability, dolomite usually had a lower permeability and porosity, and the structure of the rock was relatively dense. The rock strength as evidenced by the measured compressive strength, that limestone is softer compared to limy dolomite and dolomite, was that the proppant was more likely to penetrate into the limestone after hydraulic fracturing, giving it a relatively high depth of entry. Therefore, Group W had the highest embedding depth and Group T had a relatively low embedding depth. Group M had an embedding depth between it because it contained both dolomite and limestone.

**Table 3:** Embedding Depth and Deformation of Groups in the Propped Hydraulic Fracturing Stimulation Types

groups	types of rocks	serial number	particle size of proppant (mesh)	embedding depth (mm)	crushing and elastic deformation (mm)
T	dolomite	T1	40/70	0.019	0.87
		T2	70/140	0.11	0.84
M	limy dolomite	M1	40/70	0.024	0.87
		M2	70/140	0.14	0.81
W	limestone	W1	40/70	0.031	0.85
		W2	70/140	0.15	0.8

**Figure 11:**
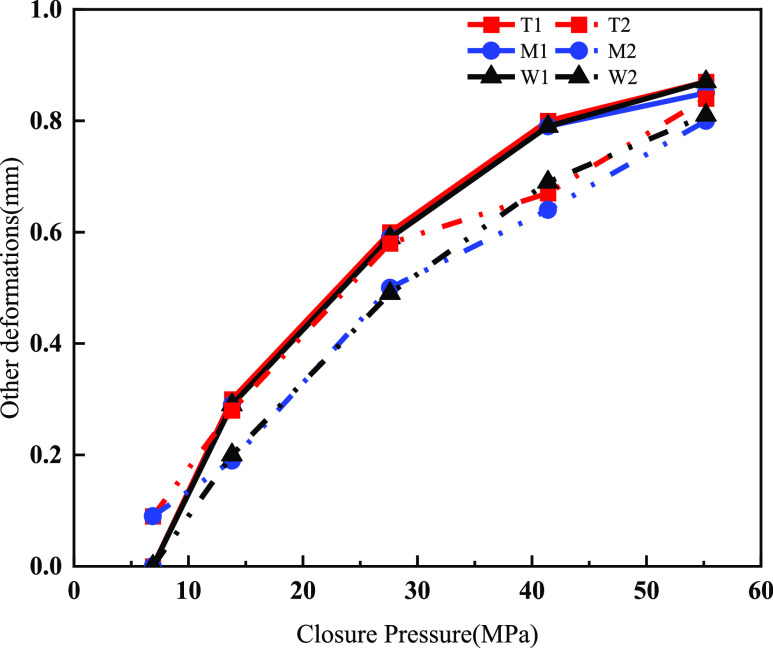
Curve of overall deformation vs closure pressure.

Dolomite was structurally dense, and the proppant embedded in it was more prone to crushing and elastic deformation; therefore, the average other deformation of dolomite was 0.855 mm, a higher value than that of limy dolomite and limestone. The larger the proppant grain size, the more significant the deformation of the cracks. The dolomite of group T had a deformation of 0.67 mm (70/140 mesh) under a pressure of 41.4 MPa. However, the deformation increased to 0.8 mm at a 40/70 mesh large grain size. The overall deformation of the cracks of the support increased with the increase in the closure pressure. The average overall deformation increased from 0.09 to 0.84 mm when the closure pressure increased from 6.9 to 55.2 MPa (Figure 11).

#### 3.1.2 Fracture Conductivity

**Figure 12:**
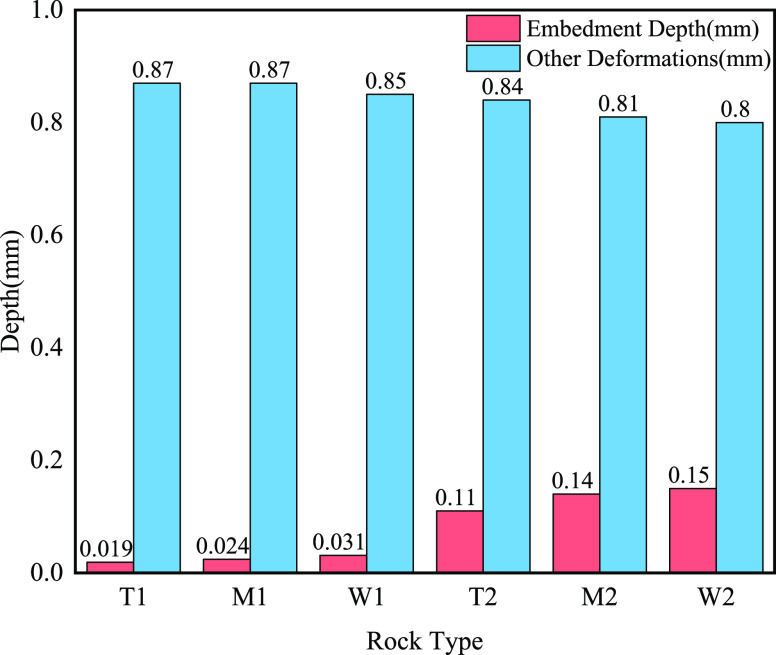
Embedding vs crushing and elastic deformation.

Group G was a comparison group of the steel plates. Figure 12 demonstrates the fracture conductivity of hydraulically fractured proppant fractures in Group T dolomite, Group M limy dolomite, Group W limestone, and the steel plate of Group G as the comparison group. The results showed that Group G had a higher fracture conductivity than the other three groups in both cases of 40/70 mesh and 70/140mu. This was because steel plates were usually made of metallic materials (e.g., steel), had high strength and hardness, and had relatively smooth surfaces. In contrast, the surface of rock slabs that had been hydraulically fractured typically had a roughness that increased resistance to the flow of liquids or gases. Therefore, the three groups of rock slabs had lower fracture conductivity than did steel plates.

**Figure 13:**
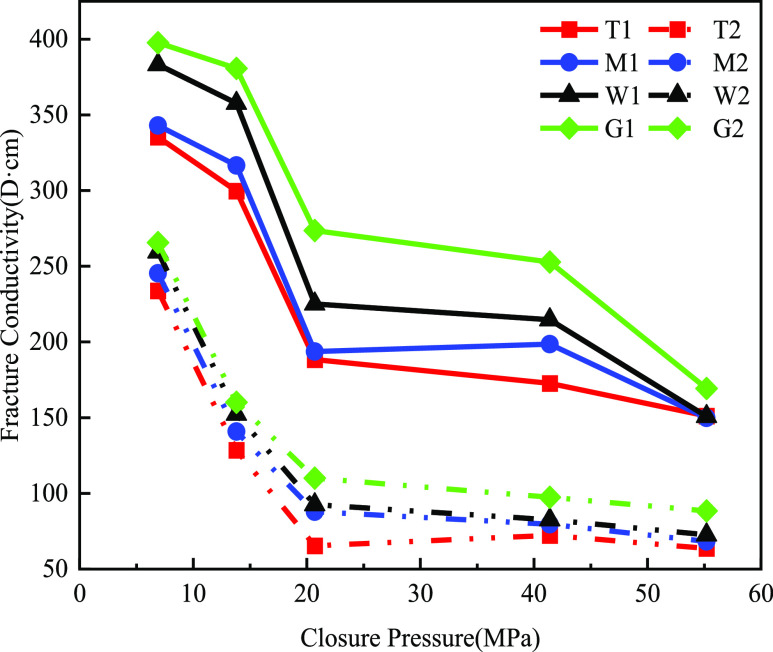
Fracture conductivity of different layers of the supporting fracture (40/70 mesh in solid line, 70/140 mesh in dotted line).

The results are shown in Figure 13. After experiments with multilayers of proppant at a proppant concentration of 5 kg/m^2^, the fracture conductivity using 40/70 mesh size ceramic was significantly higher than that using 70/140 mesh size ceramic. The fracture conductivity was larger when the large proppant was embedded. The fracture conductivity of Group T dolomite due to embedding a larger proppant (40/70 mesh) at a pressure of closure of 55.2 MPa was 151.17 MPa. The fracture conductivity of the small proppant (70/140 mesh) was 63.64 MPa, which was less than half of that of the large proppant. This was due to the large gaps among the particles of the large proppant, which created wider channels for the fluid to pass through. In contrast, small particle-sized proppant particles had smaller gaps between them and narrower channels, resulting in increased resistance to fluid passage.

The increased pore connectivity meant that liquid could flow more freely through the rock, leading to a greater hydraulic conductivity. Under the propped hydraulic fracturing stimulation approach, dolomite had a lower hydraulic conductivity than limestone for both the 40/70 mesh and 70/140 mesh grain sizes. As the closure pressure increased, all three rocks showed a greater loss of fracture conductivity. The average fracture conductivity of the three rock types was 353.83 D·cm at 6.9 MPa. At 55.2 MPa, the average fracture conductivity decreased to 150.59 D·cm (40/70 mesh). In the case of using 40/70 mesh ceramic, the fracture conductivity decreased more rapidly between 13.8 and 20.7 MPa, but the decrease of fracture conductivity gradually slowed down with the increase of closure pressure. In the case of 70/140 mesh ceramic, the fracture conductivity decreased more rapidly between 6.9 and 13.8 MPa, and then the rate gradually slowed down.

### 3.2 Acid Fracturing

#### 3.2.1 Surface Morphology Analysis

Through several experiments, we investigated the effects of the hydrochloric acid concentration and the rock type on conductivity. In these tests, we injected acid into the fracture, which led to uneven etching on the fracture surface, forming four typical etching morphology rock slabs, including groove-like etching, abutment-like etching, line-like etching, and uniform etching (Table 4). Among them, groove-like etching was manifested in the formation of dominant oil and gas seepage channels on the fracture surface, which was conducive to the conductivity of the fracture; abutment-like etching was manifested in the etching of some areas of the fracture surface, while other parts of the fracture surface formed support, the shape was similar to a bridge abutment, which was also conducive to the capacity of the conductivity; linear etching was manifested in the form of line-like etching, which was unfavorable for the capacity of the conductivity; homogeneous etching was that the fracture surface was uniformly eroded by the acid and the closure of the fracture surface was closed. Under pressure, the crack surface tended to be closed, which was unfavorable for the fracture conductivity.

**Table 4:**
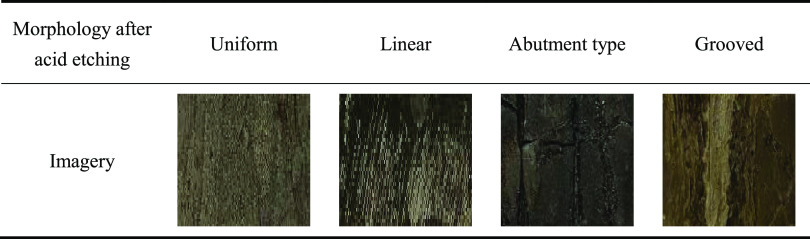
Morphology of Rock Slabs after Four Types of Acid Etching (Photograph Courtesy of Xiaojie Xia. Copyright 2023)

**Figure 14:**
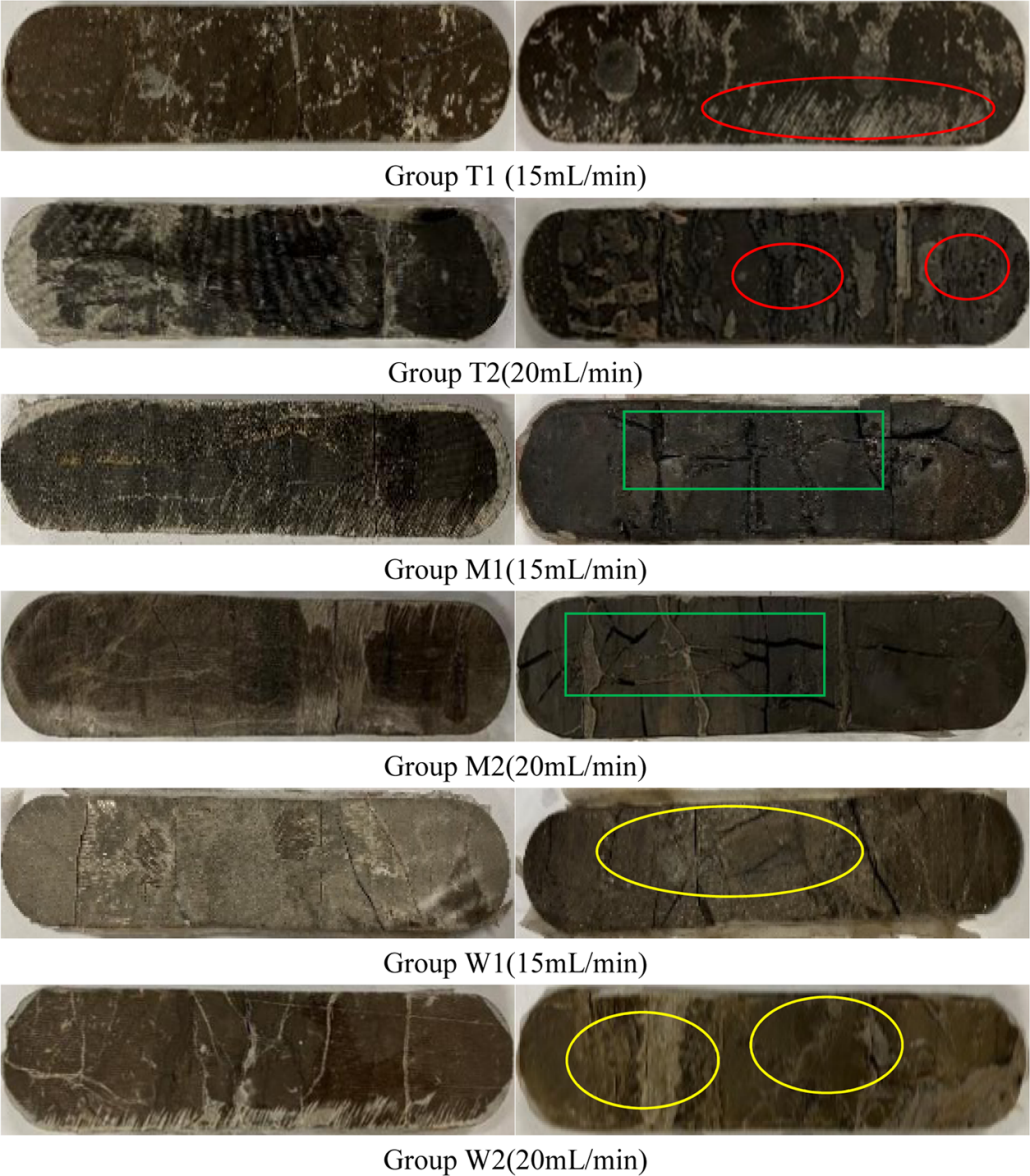
Comparison between groups of rock slabs before (left) and after (right) acid etching (Photograph courtesy of Xiaojie xia. Copyright 2023).

According to the observation, the morphology comparison results after acid etching in different layers and under different conditions are shown in Figure 14. By comparing the results of acid etching experiments with the injection volume of 15 and 20 mL/min respectively, it was found that the larger the injection volume, the more obvious etching morphology, i.e., the increased ability of inflow. Specific observations were as follows: the composition of surface minerals of group T dolomite slabs was more homogeneous, which led to the difficulty of nonuniform etching on the wall of the cracks, and the degree of nonuniform etching was poorer. Group T1 slabs showed linear etching when the injection rate was 15 mL/min, and group T2 slabs showed uniform etching when the injection rate was 20 mL/min, and there were no obvious etched grooves and support points.

This was because the limestone contained very high mineral homogeneity (dolomite content was more than 90%), and compared with other rock samples with more mineral content, the limestone did easily form the grooves and other fissures conducive to conduction of acid etching patterns. In the M group of limy dolomite, the surface morphology of the surface showed abutment-like erosion after acid etching. This was because the distribution of dolomite and limestone in this rock sample was interspersed, and the reaction rate of acid with limestone was significantly faster than that with dolomite, resulting in an asynchronous acid–rock reaction rate on the surface. Under the condition of closing pressure, the dolomite part, which reacted slower with the acid, formed a bridge pier-like support, while the dolomite part, which reacted faster with the acid, formed an oil and gas flow channel. In the limestone of group W, the reaction rate of the rock to the acid was quick and more strongly to acids, and the etching pattern was mainly dominated by the groove-type etching. The degree of nonuniform etching of groups M and W was higher than that of group T, which was more conducive to conductivity.

To summarize, the acid-etching pattern under different rock types and experimental conditions had a significant effect on the fracture conductivity. The experimental results showed that compared with the W-group limestone, the acid rock reaction speed of T-group dolomite was slower and the degree of etching was lower at the beginning, which mainly dissolved along the fracture surface. The use of large-displacement acid was conducive to deeper acid etching of the joint fracture surface, thus communicating the matrix with the fracture; at the same time, large-displacement acid could also more fully etch the wall surface of the acid-pressure fracture, and subsequently increase the degree of nonuniform etching. In acid fracturing reforming of carbonate reservoirs, the creep properties of rocks under high stress of closure needed to be considered. Additionally, the understanding of acid fracturing differences between pore-type dolomite reservoirs and fractured dolomite reserves necessitates more in-depth research.

#### 3.2.2 Three-Dimensional Scanning Analysis

**Figure 15:**
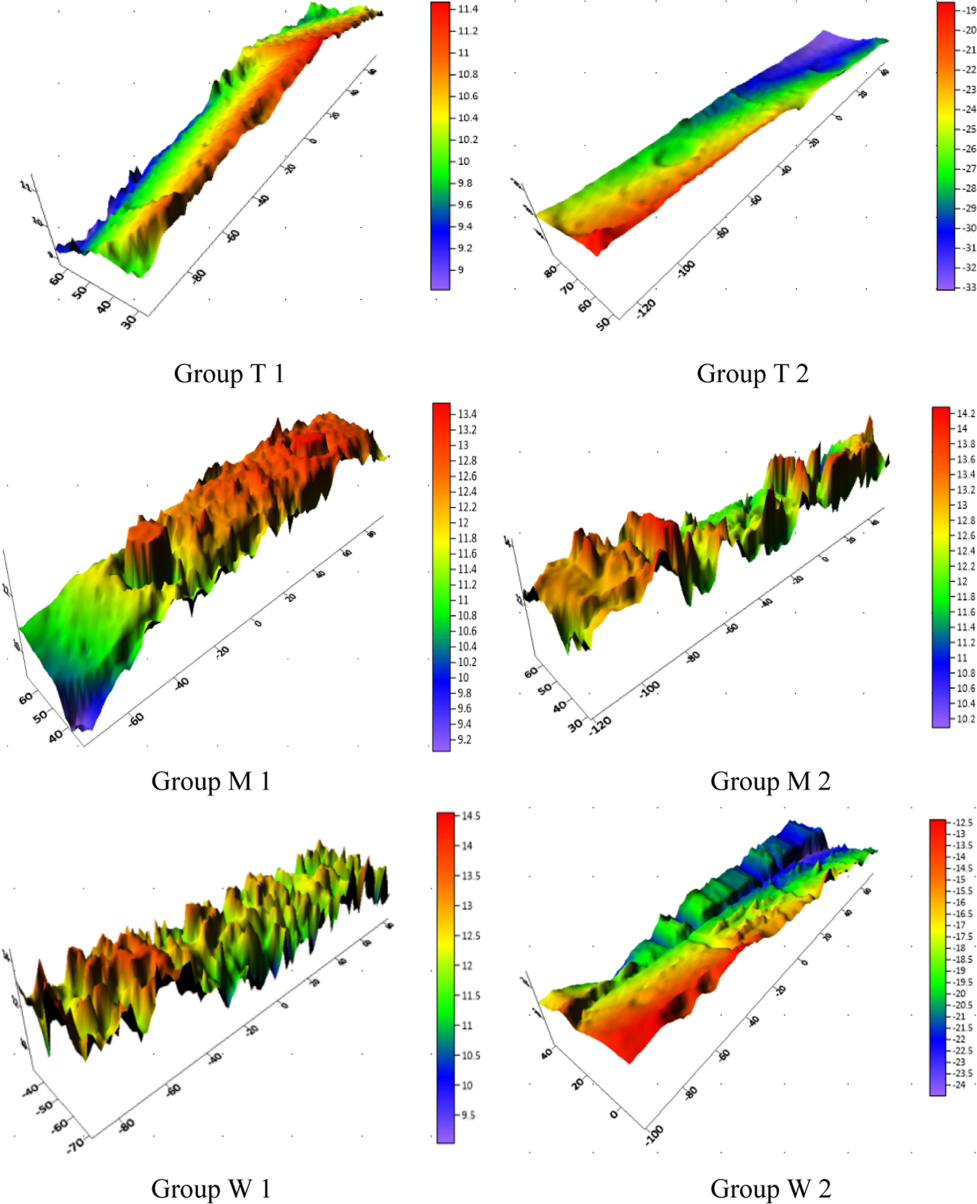
Three-dimensional images of each group of laser scans.

As shown in Figure 15, under a temperature of 106 °C and an acid mass fraction of 20%, the T group was relatively smooth, while the crack surfaces of groups M and W were relatively rough. The dolomite rock plate of group T formed uniform etching, and the crack surface was relatively smooth and had a certain conduction ability. But with the increase of closure pressure, the conduction ability decreased obviously. The limy dolomite rock plate of group M formed a more pier-like etching, and the undulation of the crack surface was obvious. The limestone rock plate of group W formed more grooves, with a larger area of support and high compressive strength, and its own nonuniform etching degree made it still have a certain conduction ability after being pressurized. However, the fracture conductivity decreased slowly with the increase in closure pressure.

#### 3.2.3 Quantitative Analysis of Etching Patterns

##### 3.2.3.1 Crack Surface Roughness

For the crack surface, many curves of crack could be found along the *x* or *y*-axis direction, and each curve could be obtained by calculating the root mean square of the *z*-axis coordinates. The average root mean square of these curves, which was used as the root-mean-square coordinates in the *z*-axis direction overall surface.^38^ It was assumed that the various discrete points in the *x* and *y* directions were uniformly divided, and the following relation could be obtained from this equation.
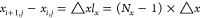
2
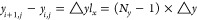
3

Tse and Cruden’s study (eq 4) on the discretization of Barton’s standard curve showed that the crack roughness coefficient JRC satisfied the equation in relation to the root-mean-square *Z*_2_.
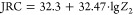
4

The relationship between the root-mean-square of the coordinates *Z*_2_ and the roughness coefficient of each discrete point in (eq 4) was used to find the JRC.

##### 3.2.3.2 Centerline Average

In that paper, not only was the roughness coefficient used to characterize the roughness of the crack surface but also the centerline means, the root-mean-square deviation of the profile, and the maximum distance between peaks and valleys were used together to describe the changes in the crack surface before and after acid pressing. This allowed for a quantitative description of the trend of the crack surface. The means of the centerline mean value represented the arithmetic square value of the height of each point on the acid-etched crack surface in the overall range, which reflected the average level of the overall height of the crack.^39^
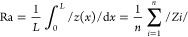
5

##### 3.2.3.3 Root-Mean-Square Deviation of Crack Profile

In that study, wavelet analysis was commonly used in physics to analyze data noise, but it was used to represent the distribution of bumps on the crack wall.^40^ The distribution was calculated as follows
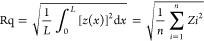
6

##### 3.2.3.4 Maximum Peak-to-Valley Distance

The formula reflected the maximum amount of undulation of the crack wall profile as follows
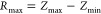
7

##### 3.2.3.5 Comparative Analysis of Acid Etching Roughness

Based on Tse and Cruden’s discretization theory and previous research results, the JRC of a three-dimensional crack surface was calculated using the root-mean-square (RMS) *Z*_2_ of discrete points after the crack had been discretized. An experiment was conducted with the help of these results. These parameters were mainly obtained by scanning the rocks after acid fracturing by a laser and computer software to identify the deformed area of these rocks. The corresponding formulas of the article were applied, and the four results of fracture surface roughness, centerline average, root-mean-square deviation of fracture profile, and maximum peak-to-valley distance were calculated.

Through the results in Table 5, it was found that the roughness coefficient of each rock slab did not differ much, and the higher the dolomite content, the smaller the roughness coefficient and the larger the maximum peak-to-valley distance. The roughness coefficient of T-group dolomite was 66.03, which was smaller than the roughness coefficient of W-group limestone, which was 67.78. The maximum peak-to-valley distance of the dolomite of group T was 7.91, which was much larger than that of the dolomite of group W, which was 3.48.

**Table 5:** Calculation Table for 3D Scanning Data Analysis

etching type	grooved	abutment type	linear	uniform
roughness coefficient	67.78	67.67	66.84	66.03
centerline average (mm)	40.08	12.35	11.66	11.94
contour root mean square difference (mm)	12.49	12.37	11.7	11.03
maximum peak-to-peak-to-valley distance (mm)	3.48	5.29	7.09	7.91

#### 3.2.4 Fracture Conductivity

**Figure 16:**
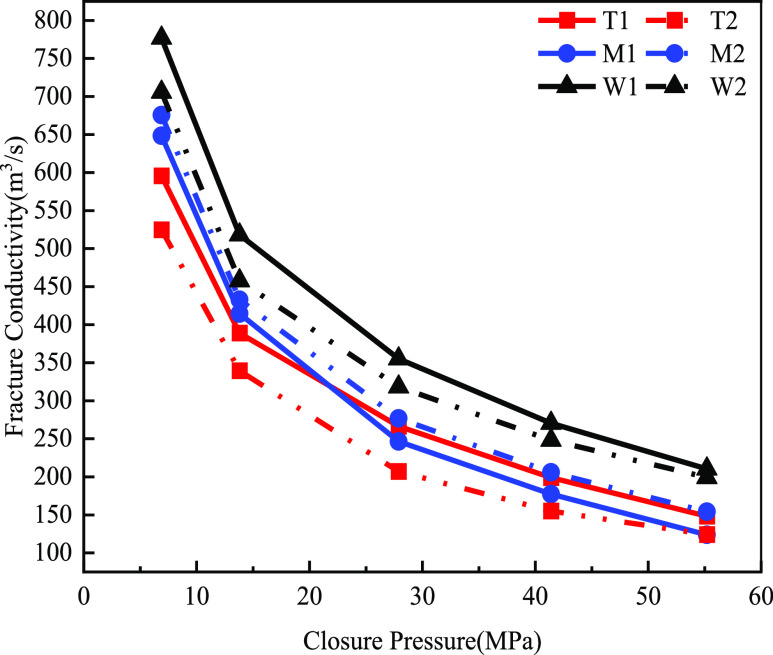
Map of fracture conductivity of different layers of acid fracturing fractures (20 mL/min in solid line, 15 mL/min in dotted line).

Compared with propped hydraulic fracturing, acid fracturing stimulation could significantly improve the fracture conductivity of the three rock types. As shown in Figure 16, the dolomite of group T had less nonuniform etching due to its purer mineral composition, and there were fewer grooves on the acid-etching surface, so the fracture conductivity of the acid fracturing was 123.72 D·cm. The limy dolomite of group M had a complicated composition and the formation of abutment-like etching and the fracture conductivity of acid fracturing, which was 148.16 D·cm. The nonuniform etching of group W limestone was stronger, forming a large number of grooves, and the proppant filled these grooves after acid fracturing plus proppant, so the fracture conductivity of acid fracturing was 210.39 D·cm. Group W limestone had a significantly higher fracture conductivity than the other two groups at 15 and 20 mL/min displacement because of more pronounced groove-type etchings.

Group T dolomite had the lowest fracture conductivity because of homogeneous and lineal etchings. Under the acidic fracturing stimulation, the fracture conductivity of the three groups of rocks decreased uniformly with the increase of closure pressure at 15 and 20 mL/min displacement. At a displacement of 20 mL/min, Group T dolomite and Group W limestone exhibited greater fracture conductivity, while Group M limy dolomite showed the opposite trend. This divergence was likely attributed to variations in their compositions, resulting in a heightened reactivity and increased extent of corrosion at larger displacement, consequently leading to elevated fracture conductivity.

### 3.3 Acid Fracturing Plus Proppant

**Figure 17:**
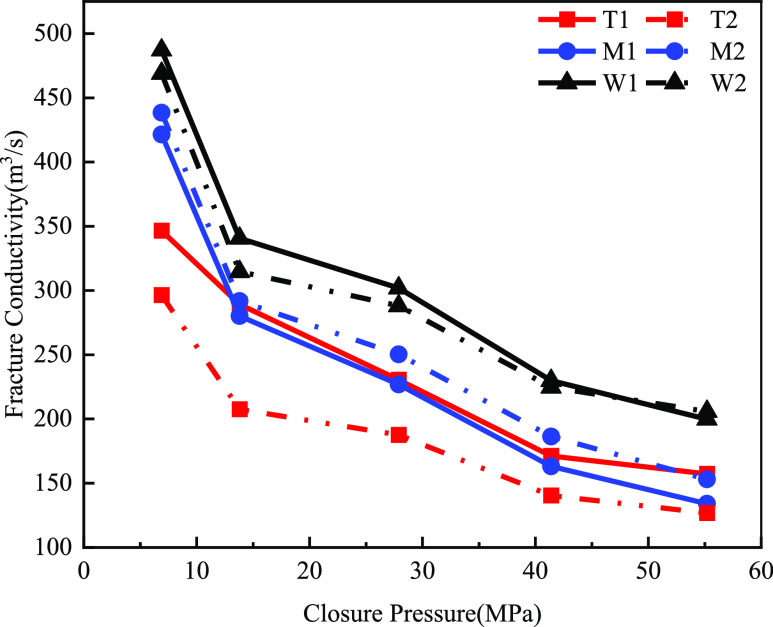
Map of fracture conductivity of different layers of acid fracturing plus proppant fractures (40/70 mesh in solid line, 70/140 mesh in dotted line).

For T group dolomite and W group limestone, the fracture conductivity using 40/70 mesh grain size was higher than 70/140 mesh grain size. This was due to the fact that the acid fracturing caused part of the uneven corrosion pattern after the proppant filling instead of blocking part of the channels. The voids with large grain sizes were easier for the fluid to pass through, resulting in higher fracture conductivity under a 40/70 mesh grain size. However, the opposite was true for the M group limy dolomite. It was possible that a problem with the experiment caused the data to be distorted. The results of comparing the acid fracturing and acid fracturing plus proppant fracture conductivity in different layers and conditions (70/140 mesh vs 40/70 mesh) are shown in Figure 17.

Table 6 shows the analysis of roughness coefficient, centerline average, root-mean-square deviation of contour, and maximal valley-to-valley distance. It was found that the roughness coefficient increased with the increase in limestone content and the fracture conductivity of acid fracturing plus proppant also increased. The roughness coefficient of W-group limestone was 67.78, which was larger than the roughness coefficient of T-group dolomite, 66.03, and the fracture conductivity was higher than that of dolomite. At the same time, the larger the maximum peak-to-valley distance, the lower the fracture conductivity of acid fracturing plus proppant. For instance, the maximum peak-to-valley distance of the dolomite of group T was 7.65, which was much larger than that of the dolomite of group W, which was 3.32. However, the fracture conductivity of acid fracturing plus proppant of group T was only 126.83 D·cm, which was smaller than that of group W, which was 199.87 D·cm. On the whole, the fracture conductivity of the dolomite of group W was higher than that of group T. The fracture conductivity of the dolomite of group M was higher than that of group T, but it was lower than that of group W.

**Table 6:** Calculation Table for the Analysis of Acid-Pressurized and Sanded 3D Scanning Data

etching type	grooved	abutment type	linear	uniform
roughness coefficient	67.78	66.83	67.93	66.94
centerline average (mm)	40.08	11.65	40.28	11.70
contour root-mean-square difference (mm)	12.49	11.67	12.60	11.74
maximum peak-to-peak-to-valley distance (mm)	3.32	5.29	5.65	7.65
fracture conductivity (D·cm)	199.87	157.26	134.01	126.83

### 3.4 Combined Conductivity Results

**Figure 18:**
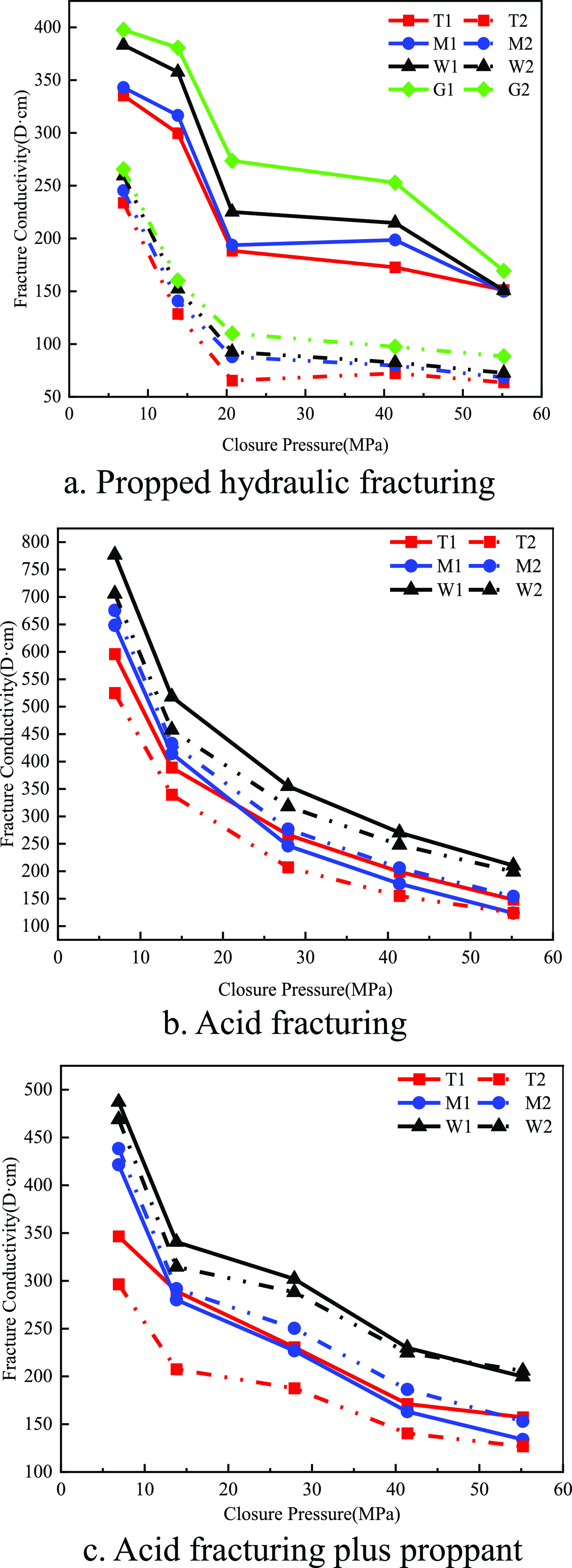
Comparison of fracture conductivity under different fracturing methods at various layers (40/70 mesh in solid line, 70/140 mesh in dotted line).

The propped hydraulic fracturing fracture conductivity under different layers, different pressures of closure, and different proppant embedding sizes are shown in Figure 18. The T-group dolomite had slightly lower fracture conductivity than the other two layers, and the smaller the proppant size and the larger the embedding depth, the more the fracture conductivity was affected, but the smaller the inflow capacity. The other two formations did not have significant differences in fracture conductivity under different proppant embedding conditions.

The loss of fracture conductivity of the 40/70 mesh ceramic of group T was greater. The loss of the fracture conductivity of group W was relatively low. The results after acid fracturing showed that the presence of channels in the fractures could significantly improve the fracture conductivity. Regardless of the layers, the fracture conductivity was significantly increased after acid fracturing. At the initial stress of closure of 6.9 MPa, the fracture conductivity of the layers was high, which was due to the faster rate of reaction of hydrochloric acid with carbonate rock, resulting in a larger amount of dissolution. Therefore, at lower pressure of closure, the rock slab cracks were larger, and the fracture conductivity was good. However, as the closure pressure increased, the fracture surface closed rapidly, resulting in a sharp decrease in the fracture conductivity of the acid-eroded cracks. When the pressure of closure of the three formations reached the final target value of 55.2 MPa, the fracture conductivity was similar. Groups M and W were close to each other, and both of them were higher than that of group T. Under the stratigraphic closure pressure, the rankings of the fracture conductivity of acid-etched fractures were limestone of group W > limy dolomite of group M > dolomite of group T. Therefore, from the point of view of fracture conductivity, the fracture-etching morphology showed a trend of change from better to worse, as follows: groove-type etching, abutment-type etching, linear-type etching, and uniform-type etching.

At larger displacement, with the increase of closure stress, the corrosion increased, the fracture grooves and support points were obvious, the fracture morphology was well distributed, and the fracture conductivity was enhanced. The fracture conductivities of Group T dolomite and Group W limestone gradually became close to each other with an increase of closure stress, but the difference between the two displacements of Group M limy dolomite was increased. However, the fracture conductivity of M-group limy dolomite was lower than that of a small injection of 15 mL/min, when the hydrochloric acid injection was 20 mL/min. This was due to the slower reaction of the acid with the rock at the small injection, which led to a longer extension of the fracture surface. In addition, the limy dolomite of group M showed a better degree of nonuniform etching at small displacement due to the nonuniform reaction of acid with the cracks, resulting in a higher fracture conductivity.

After acid fracturing plus proppant, the fracture conductivity decreased significantly with the increase in closure pressure. At a lower pressure of closure, Groups M and W showed better fracture conductivity, which was a result of the combined effect of acid fracturing plus proppant. However, their advantages diminished at a higher pressure of closure because the proppant filled the nonuniformly etched area and was compacted, reducing the synergistic effect of the two.

**Figure 19:**
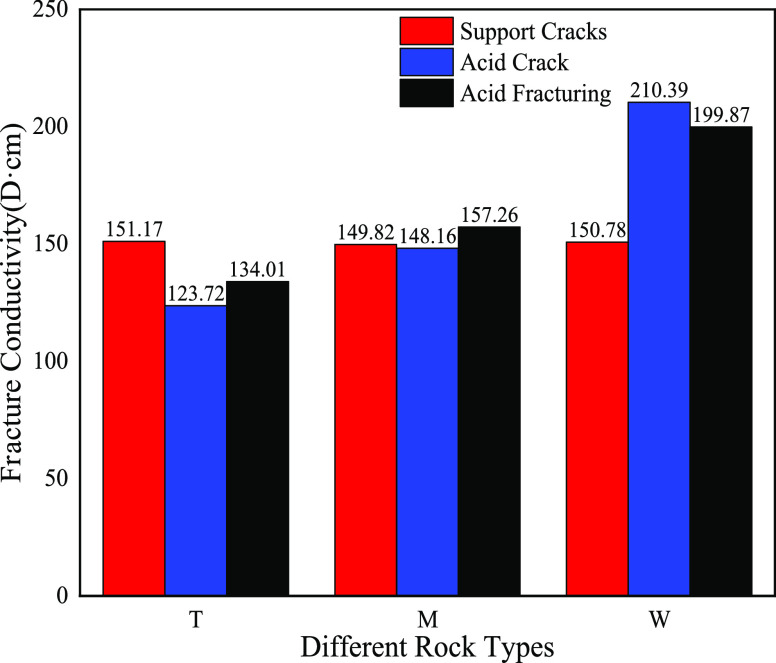
Comparison of fracture conductivities of different rock types under the target formation pressure of 55.2 MPa.

As shown in Figure 19, the actual pressure of formation was 55.2 MPa in the target reservoir. For the dolomite-type rock, the propped hydraulic fracturing method had the highest fracture conductivity, which reached 151.17 D·cm. For the limy dolomite-type rock, the acid fracturing nonuniform etching experiment had the highest fracture conductivity, which was 157.26 D·cm. For limestone-type rocks, the acid fracturing experiment followed by the acid fracturing plus proppant had the highest fracture conductivity of 210.39 D·cm.

## 4 Conclusions

(1) According to experimental results, the proppant embedding depth was larger using 70/140 mesh size, but the fracture conductivity was lower than the case of using 40/70 mesh size. The larger the proppant grain size, the more significant the deformation of the cracks. After propped hydraulic fracturing, the embedding of grain size and proppant had a great influence on the fracture conductivity. The fracture conductivity was larger when the large-size proppant was embedded.
(2) After acid fracturing and acid fracturing plus proppant, the acid-etching pattern of each group of rocks was more obvious when using a large displacement of 20 mL/min. The dolomite of group T had less nonuniform etching due to its purer mineral composition, and there were fewer grooves on the acid-etching surface. The limy dolomite of group M had a complicated composition and the formation of abutment-like etching. The nonuniform etching of group W limestone was stronger, forming a large number of grooves, and the proppant filled these grooves after acid fracturing plus proppant, so the fracture conductivity of acid fracturing plus proppant was lower than the fracture conductivity of acid fracturing only. After acid fracturing and acid fracturing plus proppant, the fracture conductivity of group M and group W were closer and both were higher than that of group T.
(3) The roughness coefficient increased with the increase in limestone content, and the fracture conductivity of acid-cemented cracks also increased. The roughness coefficient of W-group limestone was 67.78, which was larger than the roughness coefficient of T-group dolomite 66.03, and the fracture conductivity was higher than that of dolomite. At the same time, the larger the maximum peak-to-valley distance, the lower the fracture conductivity of acid fracturing and acid fracturing plus proppant.
(4) For the dolomite-type rock, the propped hydraulic fracturing method had the highest fracture conductivity, which reached 151.17 D·cm. For the limy dolomite-type rock, the acid fracturing plus proppant experiment had the highest fracture conductivity, which was 157.26 D·cm. For limestone-type rocks, the acid fracturing experiment had the highest fracture conductivity of 210.39 D·cm.

In that experiment, different fracturing methods did have different effects on the fracture conductivity of different types of rocks. However, it is also noted that the experiment has some limitations. Although different rock types are considered, in fact, even rocks of the same type may have great differences in lithology; e.g., the acid fracturing stimulation effect of a voided dolomite reservoir may be very different from that of a fractured dolomite reservoir. Therefore, it will be necessary to explore these differences in depth in subsequent studies.

## Nomenclature

*h*: = test embedment depth of a single rock slab
*h*_1_: = embedment depths of the upper slabs
*h*_2_: = lower slabs
*L*: = groove’s proppant length
*R*: = propping radius of the particles
*l_x_*, *l_y_*: = horizontal length of the crack surface in *x* or *y* direction
*z_i_*, *z*_*i*+1_: = coordinates in the *z*-axis direction of the height of the bulge of the discrete point of the crack at point *i* and point *i* + 1
*x_i_*, *x*_*i*+1_: = coordinates in the *x*-axis direction of the discrete points of the crack at point *i* and point *i* + 1
*y_i_*, *y*_*i*+1_: = horizontal coordinates in the *y*-axis direction of the discrete points of the crack at point *i* and point *i* + 1
*N_x_*, *N_y_*: = number of fine view planes of cracks along the *x* or *y* axis direction
Δ*x*, Δ*y*: = length of the projected side of the fine view plane on the *xy* plane
